# Retroperitoneal myxoid liposarcoma in a 3.5-year-old Afghan child: a case report and review of the literature

**DOI:** 10.1093/omcr/omag091

**Published:** 2026-06-08

**Authors:** Turyalai Hakimi, Sultan Ahmad Halimi

**Affiliations:** Department of Pediatric Surgery, Kabul University of Medical Science, Maiwand Teaching Hospital, Kabul, Afghanistan; Department of Pathology, Kabul University of Medical Science, Ali Abad Teaching Hospital, Kabul, Afghanistan

**Keywords:** myxoid, liposarcoma, abdominal, laparotomy, chemotherapy, cyclophosphamide

## Abstract

Myxoid liposarcoma is a malignant soft tissue sarcoma of adipocytic origin that predominantly affects adolescents and young adults. It is exceptionally rare in early childhood, with very few reported cases in children under the age of five. We present a rare case of a 3.5-year-old male child who was admitted to our emergency unit with a large abdominal mass. The patient underwent an exploratory laparotomy with complete excision of the mass, achieving negative surgical margins. Histopathological analysis confirmed the diagnosis of well-differentiated myxoid liposarcoma. The patient subsequently received six cycles of postoperative cyclophosphamide chemotherapy, administered at 20-day intervals. Follow-up tomography scan confirmed a favorable outcome with no evidence of recurrence.

## Introduction

Liposarcoma is an uncommon malignancy in the pediatric population and does not exhibit a gender predilection. According to the World Health Organization (WHO) classification, the major histological subtypes include well-differentiated, dedifferentiated, myxoid, and pleomorphic liposarcomas [1]. The etiology of the majority of soft tissue tumors remains unclear. However, in a small subset of cases—estimated at less than 10%—factors such as genetic predisposition, environmental exposures, prior radiation therapy, viral infections, and immunodeficiency have been implicated in their pathogenesis [2]. Given the extreme rarity of myxoid liposarcoma (MLS) in children and adolescents, the clinicopathological characteristics, imaging features, optimal treatment strategies, and appropriate post-treatment surveillance protocols remain poorly defined [3].

## Case report

A 3.5-year-old male child was admitted to our pediatric surgery unit with complaints of a large abdominal mass. Two months prior to admission, the mother noticed a small, asymptomatic mass in the child’s abdomen. Over the subsequent six weeks, the mass demonstrated a marked and rapid enlargement. The patient was delivered via spontaneous vaginal delivery. Notably, there was a positive third-degree family history of malignancy, as well as a history of consanguinity between the parents. On initial physical examination, the patient presented with a massive abdominal mass occupying the entire abdominal cavity, accompanied by visibly distended superficial veins radiating from the umbilicus, consistent with caput medusae (Fig. 1). Laboratory evaluation, including routine hematological and biochemical tests as well as tumor marker profiling, was performed. The results revealed anemia and evidence of a concurrent chest infection; however, serum alpha-fetoprotein (AFP) levels remained within normal limits < 5 ng/ml. Diagnostic computed tomography (CT) scan performed prior to admission revealed a large, well-defined, complex (solid and cystic) enhancing mass with lobulated margins, occupying the entire abdominal cavity. The lesion extended superiorly to the subhepatic region and inferiorly to both iliac fossae and the ureterovesical junction, confirming the presence of a large soft-tissue abdominal mass (Figs. 2 and 3).

**Figure 1:**
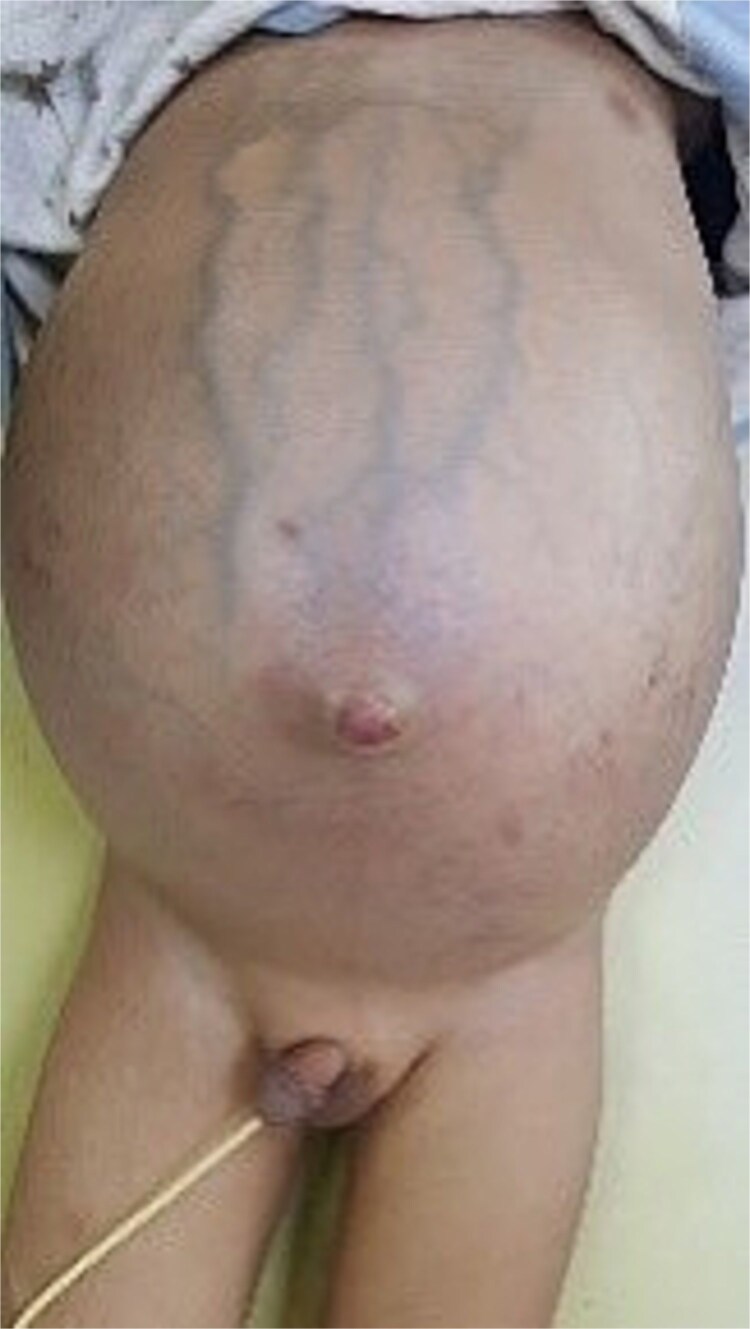
Marked abdominal distension caused by a voluminous intra-abdominal mass, with conspicuous engorgement of subcutaneous abdominal wall veins, characteristic of caput medusae.

**Figure 2:**
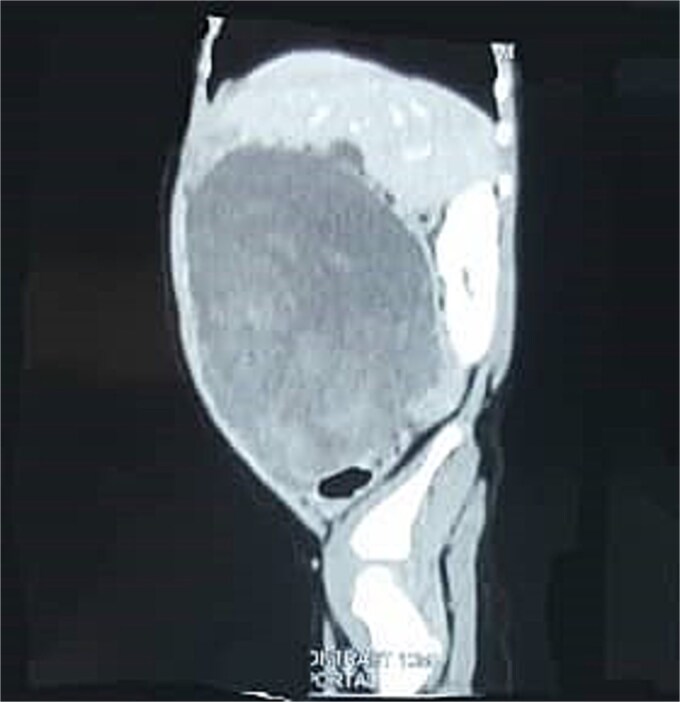
Preoperative computed tomography (CT) image showing the mass in lateral view.

**Figure 3:**
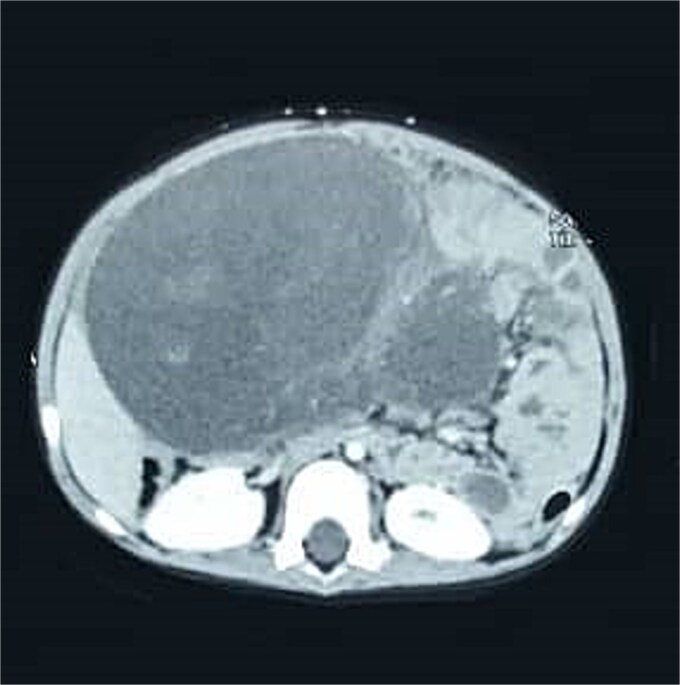
Preoperative computed tomography (CT) image showing the mass in axial view.

The lesion was accessed via a midline incision, and the mass was completely resected (Fig. 4), with negative margins achieved to the greatest extent possible. Intraoperatively, the mass was found to originate from the retrovesical region, extending throughout the entire abdominal cavity. The lesion exhibited extensive areas of hemorrhagic necrosis, underscoring its vascular and necrotic character. The abdominal catheter was removed on the third postoperative day, and the patient was discharged in stable condition following a five-day hospitalization.

**Figure 4:**
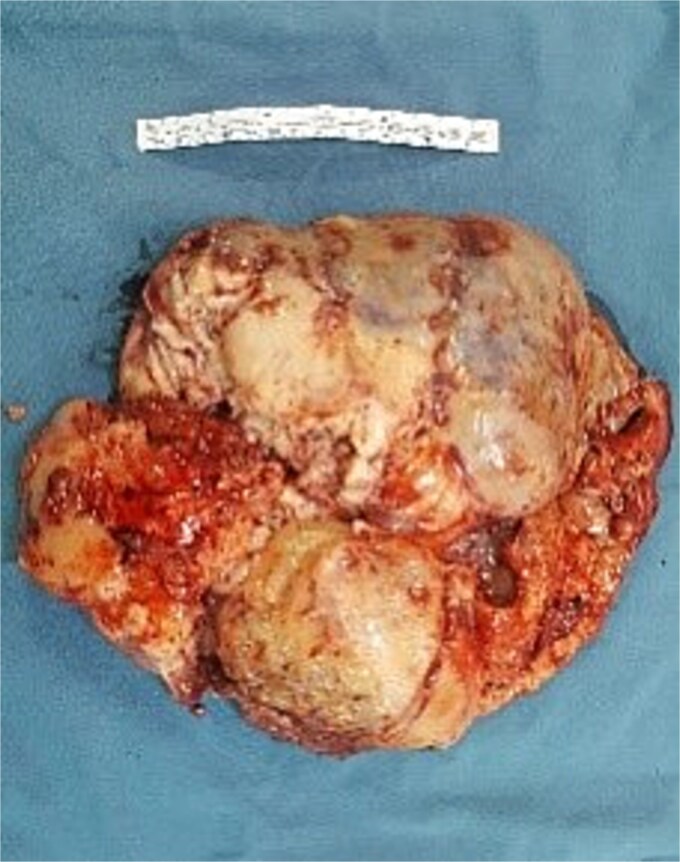
Gross specimen of the excised mass demonstrating complete surgical resection.

Histopathological analysis confirmed a diagnosis of MLS (Fig. 5). Consequently, the patient was referred to a medical oncologist and received six cycles of chemotherapy with cyclophosphamide, administered at 20-day intervals, spanning a total duration of 120 days. Upon completion of the oncologic treatment, a follow-up abdominal CT scan was performed, which demonstrated no evidence of residual disease (Fig. 6).

**Figure 5:**
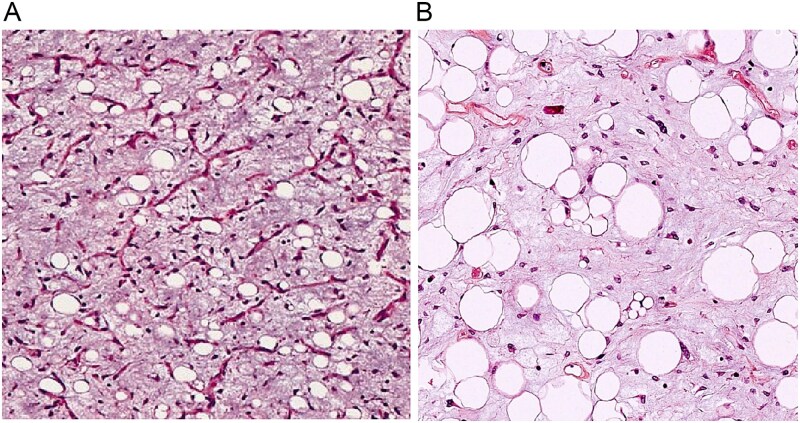
Myxoid liposarcoma. (A) Low grade myxoid liposarcoma with atypical lipocytes and numerous vascular networks. (B) Spindle cell component with nuclear atypia mixed with lipoblasts and adipocytes (20x magnification).

**Figure 6:**
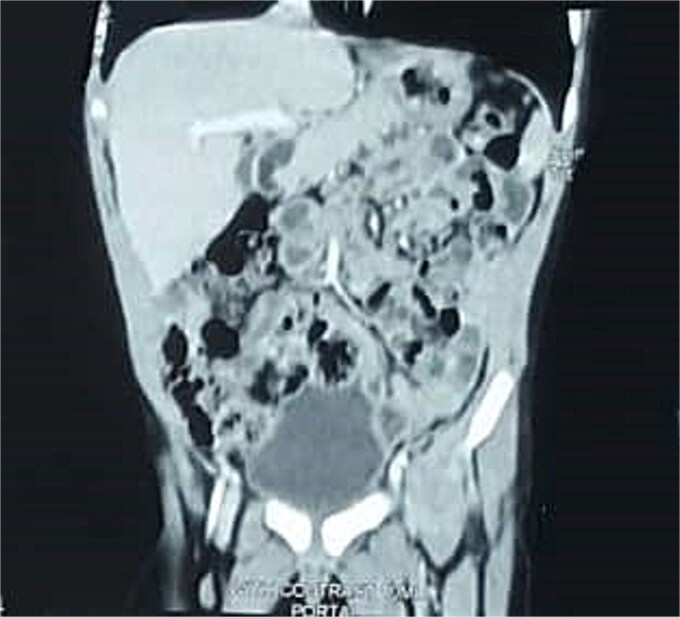
Post-operative coronal CT scan demonstrating a disease-free abdominal cavity during follow-up evaluation.

## Discussion

Morphologically, liposarcomas encompass a broad histological spectrum, ranging from low-grade neoplasms—marked by hypocellularity and a predominance of myxoid stroma—to high-grade variants that exhibit marked hypercellularity and poor differentiation, frequently characterized by a round cell component exceeding 5%, a feature previously classified under the term “round cell liposarcoma [4]. All liposarcoma subtypes typically present as large (>5 cm), deep-seated, and painless soft tissue masses [5].

MLS exhibits a peak incidence during the fourth and fifth decades of life, and its clinical behavior in adults has been extensively characterized. In contrast, the occurrence of MLS in pediatric and adolescent populations is exceedingly rare, and the clinicopathological features, imaging characteristics, optimal therapeutic approaches, and evidence-based surveillance protocols following treatment remain inadequately defined [3].

The occurrence of liposarcoma in locations such as the calf, retroperitoneum, or subcutaneous cellular tissue is exceedingly rare and has been rarely documented in the literature [6]. The evaluation of retroperitoneal liposarcoma (RPLPS) typically relies on abdominal CT or magnetic resonance imaging (MRI). These imaging modalities are instrumental in delineating the tumor’s anatomical location, dimensions, and presumed site of origin. Furthermore, they provide critical insights into the tumor’s spatial relationship with adjacent visceral and neurovascular structures, including potential compression, infiltration, or displacement. In addition, imaging facilitates the assessment of transperitoneal extension and distant metastasis. Characteristically, liposarcomas exhibit a predominantly adipose signal on both CT and MRI, aiding in their differentiation from other retroperitoneal masses [7]. In cases where patients present with distant metastases or tumors deemed unresectable, histopathological biopsy is strongly recommended to inform and guide subsequent therapeutic strategies.

Definitive surgical management of RPLPS necessitates aggressive en bloc resection aimed at achieving microscopically negative (R0) margins. In cases where localized invasion is evident, a multivisceral resection—encompassing adjacent organs such as the kidneys, intestines, surrounding adipose tissue, and musculature—is often warranted. This comprehensive surgical strategy optimizes the probability of attaining an oncologically adequate, margin-negative resection. It is crucial to carefully tailor treatment strategies, including surgery, radiotherapy, chemotherapy, targeted therapy, and immunotherapy, based on the individual characteristics of each case. Consequently, effective management necessitates a multidisciplinary approach, with close collaboration among surgical oncology, medical oncology, radiation oncology, and other relevant specialties [8].

MLS has been demonstrated to exhibit comparatively higher sensitivity to chemotherapy relative to other soft tissue sarcoma subtypes [9]. According to the 2021 guidelines of the European Society for Medical Oncology (ESMO), adjuvant chemotherapy may be considered in conjunction with wide surgical excision for chemosensitive subtypes MLS [10].

The index case involved a previously diagnosed preschool child, presenting with a large abdominal mass, who was referred to our surgical unit. The patient was managed to the best of our team’s professional capabilities with multidisciplinary approach. However, the absence of a specialized pediatric oncology center in Afghanistan posed significant challenges. Despite achieving negative surgical margins, adjuvant chemotherapy was warranted due to the presence of residual microscopic disease, evidenced by a small remnant of the tumor within the surgical field following gross total excision. Preoperative diagnostic modalities, including fine-needle aspiration cytology (FNAC) and minimally invasive image-guided sampling, were not pursued due to the unavailability of these diagnostic resources within our public hospital setting. This case exemplifies the difficulties of implementing protocol-based management in resource-constrained settings, where such approaches are often unfeasible due to the shortage of trained specialists, absence of an established referral system, limited socioeconomic resources, and inadequate healthcare infrastructure.

## Conclusion

MLS is a rare soft tissue tumor in children that requires a multidisciplinary approach involving both a pediatric surgeon and a pediatric oncologist, along with regular follow-up. Our case underscores a critical message at both the national and international levels—emphasizing the urgent need for policymakers to prioritize the establishment of specialized pediatric oncology centers. It further calls upon global entities such as the WHO and international charitable organizations to allocate dedicated funding for the development of such facilities, particularly in regions where a substantial proportion of the population consists of children, in order to improve pediatric cancer care and survival outcomes.
